# Dysbiotic salivary microbiota in dry mouth and primary Sjögren’s syndrome patients

**DOI:** 10.1371/journal.pone.0218319

**Published:** 2019-06-18

**Authors:** S. Rusthen, A. K. Kristoffersen, A. Young, H. K. Galtung, B. É. Petrovski, Ø. Palm, M. Enersen, J. L. Jensen

**Affiliations:** 1 Institute of Clinical Dentistry, Faculty of Dentistry, University of Oslo, Oslo, Norway; 2 Institute of Oral Biology, Faculty of Dentistry, University of Oslo, Oslo, Norway; 3 Department of Rheumatology, Oslo University Hospital, Oslo, Norway; University of Bergen, NORWAY

## Abstract

**Objectives:**

Primary Sjögren’s syndrome (pSS) is an autoimmune disease characterized by reduced lacrimal and salivary secretion. Sicca symptoms together with fatigue and musculoskeletal pain can significantly reduce the patients’ quality of life. Furthermore, low salivary secretion may disrupt the oral microbial homeostasis. The aim of this study was to compare the salivary microbiota from pSS patients with patients with sicca symptoms not fulfilling the classification criteria for pSS (non-SS), and with healthy controls without sicca complaints.

**Methods:**

Pellets from centrifuged chewing-stimulated whole saliva from pSS patients (n = 15), non-SS sicca patients (n = 15) and healthy controls (n = 15) were prepared. DNA was extracted and analyzed by *16S rRNA* gene sequencing. The acquired sequencing data were performed using the human oral microbiome database (HOMD).

**Results:**

We detected 42, 45, and 34 bacterial genera in saliva samples from pSS patients, non-SS sicca patients, and healthy controls, respectively. The most abundant genera in all samples were *Prevotella*, *Veillonella*, *Streptococcus*, and *Haemophilus*. At species level *Streptococcus intermedius*, *Prevotella intermedia*, *Fusobacterium nucleatum subsp*. *vincentii*, *Porphyromonas endodontalis*, *Prevotella nancensis*, *Tannerella spp*., and *Treponema spp*. were detected in the samples from pSS and non-SS only, while *Porphyromonas pasteri* was mostly found among the healthy controls.

**Conclusion:**

Our study indicated dysbiosis in the salivary microbiota from pSS and non-SS patients compared to healthy controls. Additionally, the results showed that the salivary microbiome in the pSS group differed significantly from the non-SS group.

## Introduction

Sjögren’s syndrome (SS) is an autoimmune systemic inflammatory disease that affects exocrine glands, mainly the lacrimal and salivary glands. Lymphocytic infiltration of the gland results in destruction of the tissue, loss of function, and reduced secretion of tears and saliva. The etiology of SS remains to be elucidated, although both environmental and genetic factors are believed to be involved in the pathogenesis [1]. Clinical manifestations of SS include the classical sicca symptoms of dry eyes and dry mouth, together with fatigue and musculoskeletal pain [2]. Sjögren’s syndrome may present itself as primary SS (pSS) or secondary SS (sSS) when a connective tissue disease has been diagnosed prior to the development of sicca symptoms. In 2002, the American-European Consensus Group (AECG) proposed a set of classification criteria for pSS [3,4], that includes dry mouth, dry eyes, reduced salivary secretion, reduced lacrimal secretion, presence of Ro/SSA and/or La/SSB autoantibodies, and lymphocyte infiltration in minor salivary glands. In order to be classified as pSS, four of the six criteria must be met, including a positive minor salivary gland biopsy or positive serum antibodies. Alternatively, any three of the four objective criteria should be fulfilled. Interestingly, a serological profile characterized by anti-Ro/SSA and anti-La/SSB, antibodies against extractable nuclear antigens, has been reported in 50–70% and 25–40% of adults with pSS, respectively [5]. The prevalence of pSS is reported to vary from 0.05 to 1% in the European population, depending on which classification criteria have been used [6,7]. The criteria from the AECG are well accepted and are often used in research and clinical practice [8].

Dry mouth due to reduced salivary secretion in pSS has been shown to change the microbiota of the oral cavity [9]. The composition of the microbiota may be divided into resident species (core) and transient species (variable), where those organisms that are always present represent the residents or the core microbiota [10]. Species of the core microbiota that are always present in high numbers (>1%) have been called ‘indigenous’, while those present in low numbers (<1%) are termed ‘supplemental’ species [11]. When environmental changes occur, the supplemental species may become indigenous, indicating a shift of the microbial composition [12]. Several other host factors, such as diet, oral hygiene, drugs, smoking, systemic infections, and geographical and climatic conditions, may also promote a microbial shift [10,13].

The oral microbiome includes species from different phyla, the most abundant phylum is *Firmicutes* with *Streptococcus* as one of the main genus groups with many different species [14]. Furthermore, phyla such as *Actinobacteria*, *Bacteroidetes*, *Fusobacteria*, and *Proteobacteria* are often present. *Synergistetes* and *Spirochetes* are represented in lower numbers (low abundance), but their species may nonetheless have important functions in the microbiome. The low abundance bacteria may become pathogenic by increasing in proportion, thus causing imbalance in the microbiome composition. This results in a dysregulation, called dysbiosis, that may play a role in various systemic diseases [12,13], and maybe in the pathogenesis of pSS. A possible link between gut dysbiosis, disease manifestation in pSS, and autoimmunity was demonstrated by De Paiva and co-workers [15]. Similarly, a shift of the bacterial composition in the oral cavity may trigger the development of, and cause progression and maintenance of autoimmune diseases such as pSS [16].

In order to investigate a possible dysbiosis in pSS, we aimed to compare the salivary bacterial composition in pSS with non-SS sicca patients and healthy controls.

## Materials and methods

The study population consisted of 45 female participants aged 30 to 80 years that were divided into three groups of fifteen persons. The first group was composed of patients with pSS, who fulfilled the AECG classification criteria for pSS (pSS group). The second group consisted of subjects with sicca symptoms, but without anti-SSA/SSB autoantibodies and with a negative salivary gland biopsy, thus not fulfilling the AECG criteria for pSS (non-SS group). The third group was made up of healthy persons without complaints of dry mouth or dry eyes (control group).

A comprehensive oral clinical examination of all participants was performed by calibrated dentists. The parameters registered included the total number of teeth present, the number of missing and decayed teeth, number of mobile teeth and gingivitis. Dental caries experience was recorded using the DMF-system (DMFT: the sum of the number of decayed (D), missed (M), and filled (F) teeth (T)), to illustrate the dental status of each group.

The clinical assessment of oral dryness score (CODS) [17] was used to assess objective oral dryness. CODS consists of 10 features of objective oral dryness, and is scored as 0–3 (none to mild), 4–6 (moderate), and 7–10 (severe). Subjective oral dryness was scored according to the Shortened Xerostomia Inventory (SXI) [18], which is a five statement questionnaire producing a sum score range from 5 to 15, where 15 indicates a very severely dry mouth. Mean scores for CODS, SXI and DMFT were determined for each group.

Unstimulated whole saliva (UWS) and stimulated whole saliva (SWS) samples were collected following a standardized protocol. Patients refrained from eating, drinking, and smoking one hour before their appointment. For UWS the participants were asked to swallow any saliva in the mouth, and saliva was then collected for 15 min in a pre-weighed cup kept on ice. For SWS the participants chewed on a paraffin wax tablet (Ivoclar Vivadent, Schaan, Lichtenstein) for approximately 30 s before swallowing and then they continued chewing for 5 min, expectorating saliva regularly into a pre-weighed cup kept on ice. Following sample collection, saliva secretion rate (ml/min) was calculated. Patients who had a UWS secretion rate ≤ 1.5 ml/15 min (≤ 0.1 ml/min) were categorized as suffering from hyposalivation, i.e. a documented pathological reduction in saliva secretion rate [19].

The protocol for the study was approved by the Norwegian Regional Committee for Medical and Health Research Ethics (REK 2015/363), and written informed consent was obtained from all participants. All saliva samples were initially stored at -80°C. Prior to analysis, SWS samples were defrosted on ice and centrifuged at 4000 rpm for 10 min at 4°C. The supernatant was removed and 0.5 ml of RNAlater (RNA-L; Life Technologies, Grand Island, NY, USA) was added to each saliva pellet to preserve DNA. The pellets were then stored at 4°C overnight and then moved to a 20°C for further storage.

### DNA isolation, PCR amplification, and gene sequencing

DNA extraction of the SWS samples was performed using the MasterPure DNA isolation kit from Epicentre (MCD85201, Epicentre Biotechnologies, WI, USA). The *16S rRNA* gene was amplified using universal *16S rRNA* gene primers, forward primer 334f (5’- CCAGACTCCTACGGGAGGCAGC-3’), and reverse primer 939r (5’- CTTGTGCGGGCCCCCGTCAATTC-3’) [20,21] targeting the V3-V5 hypervariable region. PCR reactions were performed with 28 cycles in 20 μl mixture of OneTaq mastermix (New England Biolabs Inc. Ipswich, MA, USA) in an Applied Biosystem PCR cycler (Thermal cycler, Foster City, CA, USA). PCR amplification was performed with an initial denaturation step of 96°C for 2 min, 28 cycles of denaturation at 96°C for 30 s, annealing at 61°C for 30 s, and elongation at 72°C for 30 s, followed by a final extension step of 72°C for 4 min and 4°C. A second PCR with the fusion adaptor primer A with *16S rRNA* 334f and index sequence, and adaptor primer B with *16S rRNA* 939r sequence was performed with initial denaturation at 96°C for 1 min, 20 cycles of denaturation at 96°C for 30 s, primer annealing at 59°C for 30 s and elongation at 72°C for 30 s and final extension at 72°C 4 min, and 4°C. Then the amplicons were purified using Agencourt Ampure Beads (Agencourt Bioscience Corporation, Beckman Coulter Company, Inc.CA, USA) followed by DNA quantitation and quality examination with a Agilent 2100 Bio analyzer and the High Sensitivity DNA Assay kit (Agilent Technologies, Santa Clara, CA, USA). The final amplicon preparation products were used in emulsion PCR via Roche GS Lib-L kit (Roche Diagnostics Gmbh, Mannheim, Germany) with the use of a molecules-per-bead ratio of 0.7. The emulsion PCR, library bead purification, and sequencing on the Roche 454 GS Junior system was performed according to the manufacturer´s instructions.

### Pyrosequencing data processing and taxonomic classification

The data analysis workflow was based on the Quantitative Insights into Microbial Ecology (QIIME 1.8.0) pipeline [22]. The pyrosequencing data sff file was demultiplexed with the command split_library.py with a restriction in read length removal of reads that are smaller than 300 bases and larger than 600 bases and homopolymer more than 6 bases. Then the command “denoise_wrapper.py” was used to remove noise and qualify the correct signaling bases in the sequence, thus increasing the accuracy of the whole QIIME pipeline. Chimera filtering was then performed with the UCHIME algorithm by the reference-based and the de novo method [23]. Reads that were classified as chimeric by both methods were removed. For clustering reads into operational taxonomic units (OTUs), each sample group was first analyzed separately with the used “pick_open_reference_otus.py” with the saliva database (ssu ref99) and the HOMD database (HOMD_16S_Ref Seq_V14.51)).The OTU diversity in each sample was analyzed by “alpha_diversity.py” with the metrics; chao1, Shannon and Simpson. The Kruskal-Wallis test was used to compare diversity between the groups.

Species diversity in each sample was determined by blasting individual sample sequences directly in the HOMD *16S rRNA* blasting tools (homd.org/) with a cutoff at 98.5%. Each species identified and included in the species figures was aligned with 99–100% identity with reads length of 380–550 nt. All sample sequences were also analyzed in the SILVAngs [24] to visualize the overview of the taxa diversity in each group and to compare them with the QIIME analysis. Roche GS junior samples from pSS, non-SS and control groups were submitted to ENA (European Nucleotide Archive).

### Statistical methods

Data analysis was performed using descriptive statistical analysis; percentage distribution, mean and standard deviation (SD). In the case of non-normality of continuous variables, median and interquartile ranges (IQR, measure of variability) and max/min ranges were also calculated. Normality of continuous variables was tested on Q-Q- plot and by the Shapiro-Wilk and Kolmogorov-Smirnov test. When the normality assumption was satisfied, the one-way ANOVA with Bonferroni post-hoc test was used to compare means of continuous- and numerical variables, otherwise Kruskal-Wallis ANOVA with Dunn`s post-hoc test was used. Homogeneity of variance was analyzed with Levene`s test (Table 1).

**Table 1:** Characteristics of the three participant groups.

Characteristics	pSS (n = 15)	non-SS (n = 15)	control (n = 15)	p-value^§^
**Age** (yr) mean±SD	53.20 ±11.93	53.07±12.04	56.00±14.59	NS
**Smoker** n (%)	3 (20.00)	3 (20.00)	0 (0.00)	NS
**Hyposalivation** (UWS≤0.1 ml min^-1^) n (%)	11 (73.33)	10 (66.67)	0 (0.00)	p = 0.0000***
**Unstimulated saliva flow rate** (ml min^-1^)				
mean±SD	0.09±0.09	0.10±0.07	0.25±0.16	p = 0.0003***
median (IQR)	0.07 (0.04–0.10) **	0.09 (0.03–0.14) **	0.2 (0.12–0.32)	
range	0.01–0.38	0.01–0.28	0.09–0.62	
**Stimulated saliva flow rate** (ml min^-1^)				
mean±SD	0.78±0.42	0.85±0.41	1.49±0.72	
median (IQR)	0.7 (0.4–1.1)**	0.83(0.55–1.23)**	1.39(1.04–1.74)	p = 0.001**
range	0.11–1.51	0.25–1.77	0.59–3.22	
**CODS**				
mean±SD	3.93±1.91	4.07±1.71	0.93±1.03	p = 0.0001***
median (IQR)	4 (3–5)***	4 (3–6)***	1 (0–2)	
range	0–7	1–6	0–3	
**SXI score**				
mean±SD	12.13±2.20	12.40±1.81	5.67±0.82	p = 0.0001***
median (IQR)	12 (10–14)***	12 (11–14)***	5 (5–6)	
range	8–15	9–15	5–7	
**Number of teeth**				
mean±SD	26±2.53	24.8±3.05	26.07±4.71	NS
median (IQR)	26 (26–28)	25 (22–28)	28 (27–28)	
range	20–28	20–28	10–28	
**DMFT**				
mean±SD	17.20±6.36	17.00±6.22	15.60±7.77	NS
**Mobile teeth** n (%)	1 (6.67)	1 (6.67)	0 (0.00)	NS
**Gingivitis** n (%)	4 (26.66)	2 (13.33)	0 (0.00)	NS
**Number of medications taken** n (%)				
none	6 (40.00)	2 (13.33)	11 (73.33)	
one	4 (26.67)	5 (33.33)	4 (26.67)	
two	5 (33.33)	5 (33.33)	0 (0.00)	0.004**
≥three	0 (0.00)	3 (20.00)	0 (0.00)	
**Last dental visit** n (%)				
<6 months	6 (40.00)	8 (53.33)	8 (53.33)	
7–12 months	6 (40.00)	6 (40.00)	4 (26.67)	NS
13–24 months	1 (6.67)	1 (6.67)	3 (20.00)	
2–5 yr	2 (13.33)	0 (0.00)	0 (0.00)	

^§^ p-values indicate that the three groups are significantly different from each other.

NS: Not Significant

*** (p<0.001) and

** (p<0.01) show the significant differences between the pSS or non-SS groups with the control

SD: Standard Deviation

AECG: American-European Consensus Group

CODS: Clinical Oral Dryness Score

SXI: Shortened Xerostomia Inventory

DMFT: Decayed, Missing and Filled Teeth

IQR: Interquartile Range

The Chi-square (*χ*^2^) and the Fisher`s exact tests were used to determine the differences in the distribution of categorical variables, while a 2-sample z-test was applied to detect the differences in the proportions of the microbial species between the studied groups. If the sample within each column was ≤1, then the z-test could not be used. The significance level was set as p<0.05 and adjusted with Bonferroni correction to p < 0.05/n (where n is the number of analyses). SPSS software (SPSS version 24, IBM, Armonk, NY, USA) was used for the statistical analyses.

## Results

### Clinical parameters

Table 1 shows the clinical characteristics of the participants. There was no significant difference between the groups with respect to age and smoking status. Saliva secretion rates (UWS and SWS) were significantly lower for the pSS and non-SS groups compared to the control group. In addition, pSS and non-SS patients had significantly higher CODS and SXI scores compared to controls (p<0.0001). There were no significant differences between the groups in the number of teeth, DMFT, number of mobile teeth and gingivitis. The number of medications used and the time since the participant last attended the dentist are also shown in Table 1.

Additional data for the pSS patients were obtained from the Department of Rheumatology, Oslo University Hospital, and the information is summarized in Table 2. In particular, all pSS patients were anti-SSA/Ro positive in order to secure a homogenous patient population.

**Table 2:** Clinical features of the pSS patients.

	pSS (n = 15)
Years since onset of symptoms (mean±SD)	9±12.3
Years since time of diagnosis (mean±SD)	4±6.7
ANA n (%)	15 (100)
SSA/Ro positive subjects n (%)	15 (100)
Ro52 n (%)	13 (87)
Ro60 n (%)	14 (93)
SSB/La n (%)	8 (53)
Rheumatoid factor (RF) n (%)	2 (13)
Elevated IgG level (>15.0 g/l) n (%)	6 (40)
Low complement C3 or C4 n (%)	4 (27)
Leucopenia (<4.0 x 10^9^/L) n (%)	4 (27)
Lyphopenia (<1.1 x 10^9^/L) n (%)	2 (13)
Swelling of parotid gland n (%)	6 (40)
Extraglandular manifestations n (%)	3 (20)
Cutaneous vasculitis n (%)	2 (13)
Arthritis n (%)	1 (7)

### Composition of the salivary microbiota at phylum level

A total of 76110 sequence reads were obtained after quality filtering with an average length of 380–550 nt. In the pSS group (n = 15 samples), the total sequence reads were 18677 (reads per sample; min = 919, max = 1751) and in the non-SS group (n = 15 samples) the total sequence reads were 39492 (reads per sample; min = 957, max = 6365). For the control group (n = 15 samples), the total sequence reads were 17941 (reads per sample; min = 868, max = 1617). The alpha diversity analyses (Chao 1, Shannon and Simpson) showed no significant differences between the three groups.

**Fig 1:**
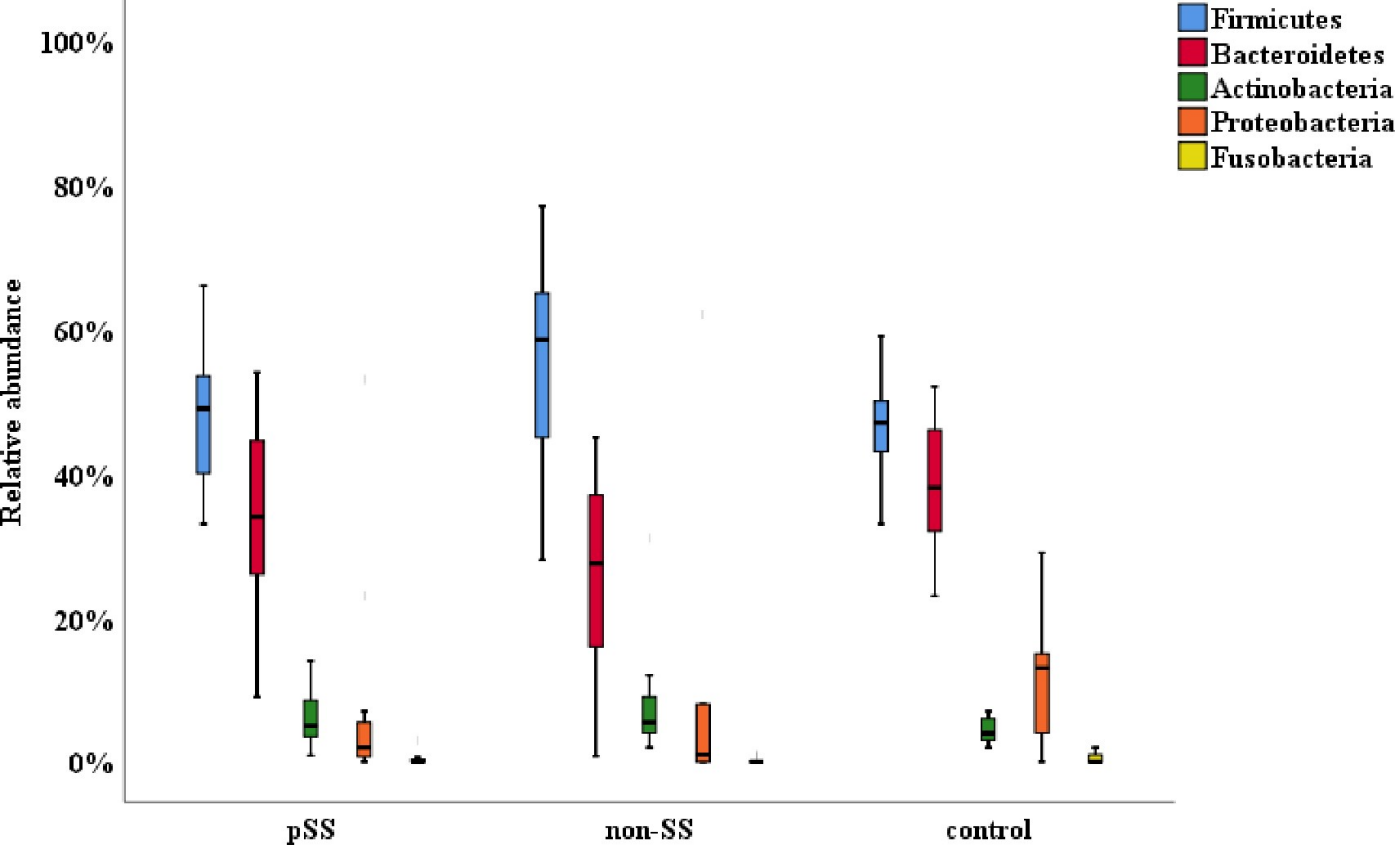
Relative abundance of the most major phyla in saliva from pSS, non-SS and control groups.

Nine different bacterial phyla were detected. The most predominant common to the three groups were *Firmicutes*, *Bacteroidetes*, *Actinobacteria*, *Proteobacteria*, and *Fusobacteria*. The relative abundances of predominant phyla are shown in Fig 1.

The most abundant phylum detected in all three groups was *Firmicutes* (for pSS, non SS and controls respectively; 50%, 59%, 48%), followed by *Bacteroidetes* (34%, 26%, 35%). *Actinobacteria* was found more abundantly in non-SS group (7.6%) than in the pSS (6.3%) and control groups (4%). *Proteobacteria* was found in higher abundance in the control group (12.7%) compared to the pSS (7.6%) and non-SS (8.5%) groups. *Fusobacteria* was found at low levels in pSS, non-SS, as well as in the control group (0.8%, 1.29%, 1.35%), respectively. There were no significant differences in the phyla abundance between the three groups.

### Composition of the salivary microbiota at genus level

**Fig 2:**
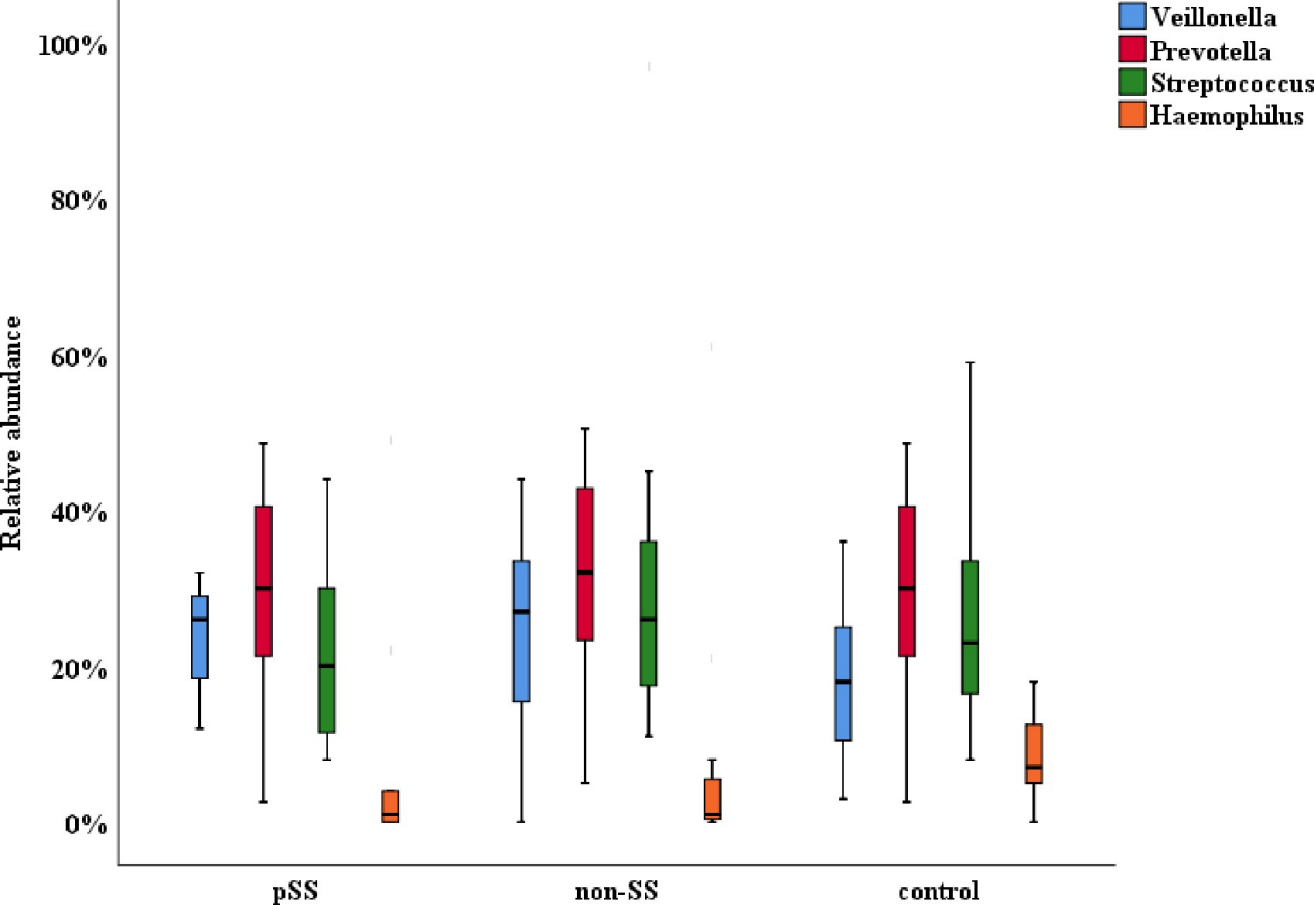
Relative abundance of the major bacterial genera in saliva from pSS, non-SS, and control groups.

Fifty-nine different bacterial genera were detected in the saliva samples, with 42, 45, and 34 different genera in the pSS, non-SS and control groups, respectively. The most abundant genera were *Prevotella* in the phylum *Bacteroidetes*, *Veillonella* and *Streptococcus* in phylum *Firmicutes*, and *Haemophilus* in phylum *Proteobacteria*, as illustrated in Fig 2.

The mean abundance of the *Prevotella* genus was higher in the non-SS group (32%) compared to the pSS group (30%) and the controls (30%). *Veillonella* was also higher in the pSS group (26%) and the non-SS (27%) compared to controls (18%). However, the mean abundance of *Streptococcus* was lower in the pSS group (20%) than in the non-SS group (26%) and the control group (23%). *Haemophilus* was also less abundant in the pSS and non-SS groups (1%) than in the control group (7%). There were no significant differences between pSS, non-SS and controls in relation to abundance of the most predominant genera. Only *Haemophilus* (p = 0.033) and *Neisseria* (p = 0.003) were significantly decreased in pSS and non-SS compared to controls.

### Composition of the salivary microbiota at species level

In total, 183 bacterial species were detected in the saliva samples investigated in this study, comprising 124, 152 and 102 species in the pSS patient group, non-SS patient group, and control group, respectively.

Some genera showed higher species diversity in the pSS and non-SS groups compared to the control group. An example of this was the number of *Prevotella* species detected in the pSS (21) and non-SS (18) groups compared to the control group (14). In contrast, some genera such as *Streptococcus* and *Neisseria* showed less species diversity in the pSS and non-SS groups compared to the control group. Only 5 different *Neisseria* species were detected in the pSS and non-SS groups compared to 12 different species in the control group. Regarding the *Streptococcus* genus group, there were small species differences with 23 and 21 different species detected in the pSS and non-SS groups, respectively, compared to 26 different species in the control group.

#### Predominant species (resident) found in all three groups

**Fig 3:**
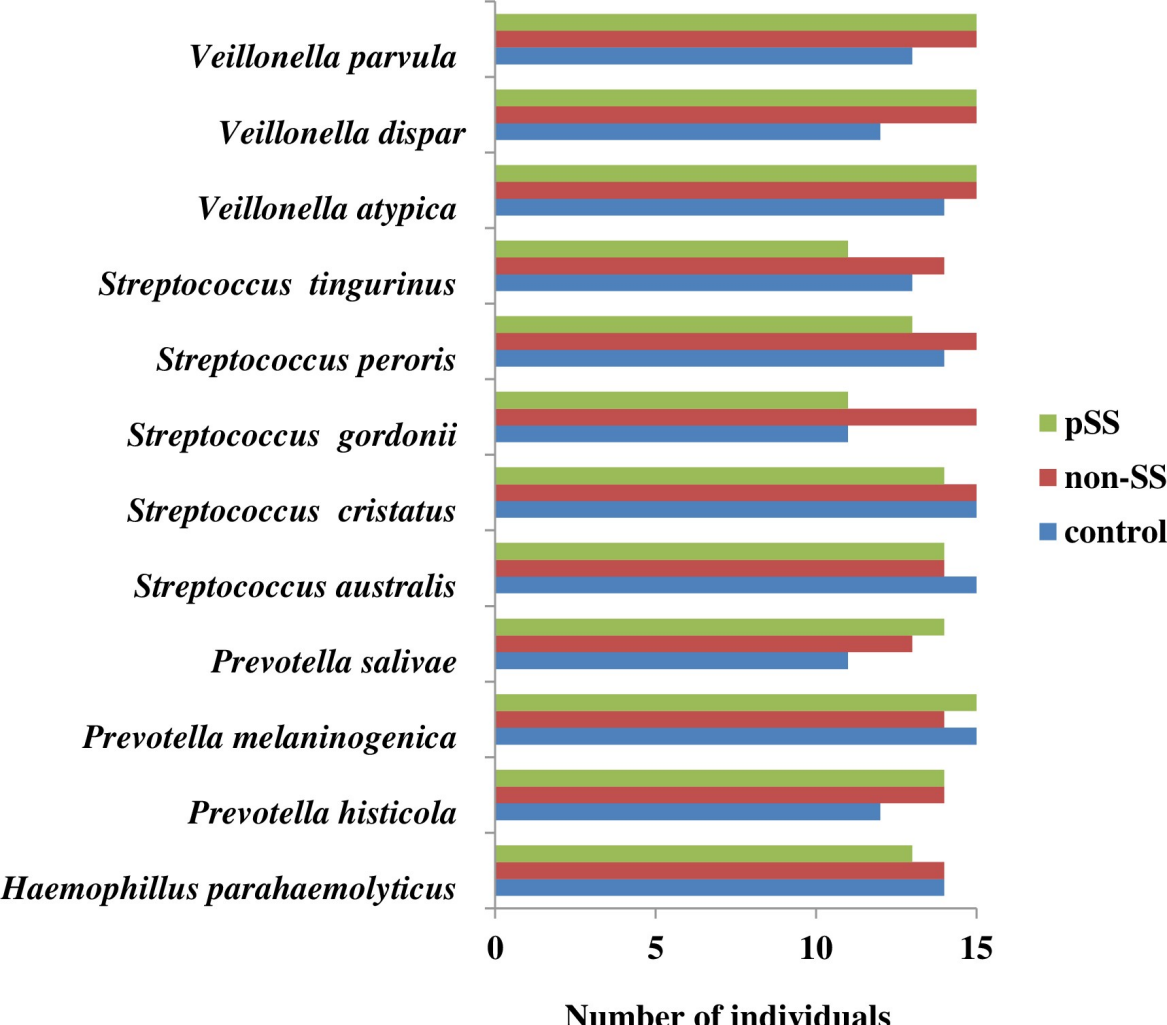
Predominant bacterial species detected in the three salivary sample groups.

Twelve main species represented a core microbiome detected in nearly all of the samples (80%-100%) in all three groups. There were three species from both *Veillonella* and *Prevotella*, five *Streptococcus* species, and one *Haemophilus* species, as illustrated in Fig 3.

#### Transient (variable) species found in the three salivary sample groups

Twenty-one species showed significant differences in their bacterial profile between the three groups, as shown in Table 3.

**Table 3:** Significant different species profiles between groups.

Bacterial species	pSS	non-SS	control	p-value^§^
	n = 15 (%)	n = 15 (%)	n = 15 (%)	
*Actinomyces lingnae*	10 (66.7)	15 (100)	4 (26.7)	0.000
*Atopobium parvulum*	10 (66.7)	14 (93.3)	6 (40.0)	0.009
*Capnocytophaga leadbetteri*	0 (0.0)	6 (40.0)	0 (0.0)	0.002
*Fusobacterium nucleatum subsp vincentii*	5 (33.3)	6 (40.0)	0 (0.0)	0.022
*Fusobacterium periodonticum*	0 (0.0)	5 (33.3)	7 (46.7)	0.008
*Granulicatella adiacens*	7 (46.7)	14 (93.3)	10 (66.7)	0.027
*Lachnoanaerobaculum orale*	6 (40.0)	6 (40.0)	0 (0.0)	0.011
*Megasphaera micronuciformis*	12 (80.0)	13 (86.7)	4 (26.7)	0.002
*Mitsuokella sp*	2 (13.3)	6 (40.0)	0 (0.0)	0.017
*Neisseria flavescens*	2 (13.3)	7 (46.7)	11 (73.3)	0.005
*Oribacterium asaccharolyticum*	3 (20.0)	7 (46.7)	0 (0.0)	0.008
*Peptostreptococcaceaex 1 G1*	0 (0.0)	6 (40.0)	2 (13.3)	0.017
*Porphyromonas pasteri*	4 (26.7)	6 (40.0)	12 (80.0)	0.01
*Prevotella nanceiensis*	8 (53.3)	3 (20.0)	0 (0.0)	0.002
*Prevotella oralis*	10 (66.7)	14 (93.3)	8 (53.3)	0.045
*Ruminococcaceae G1 sp*	0 (0.0)	6 (40.0)	0 (0.0)	0.002
*Stomatobaculum sp*	0 (0.0)	5 (33.3)	6 (40.0)	0.022
*Streptococcus mutans*	8 (53.3)	6 (40.0)	1 (6.7)	0.02
*Streptococcus parasanguinis II*	15 (100.0)	15 (100.0)	10 (66.7)	0.007
*Streptococcus salivarius*	1 (6.7)	5 (33.3)	0 (0.0)	0.035
*Streptococcus vestibularis*	14 (93.3)	15 (100.0)	10 (66.7)	0.035

^**§**^Chi-square (χ2) and Fisher`s exact test.

**Fig 4:**
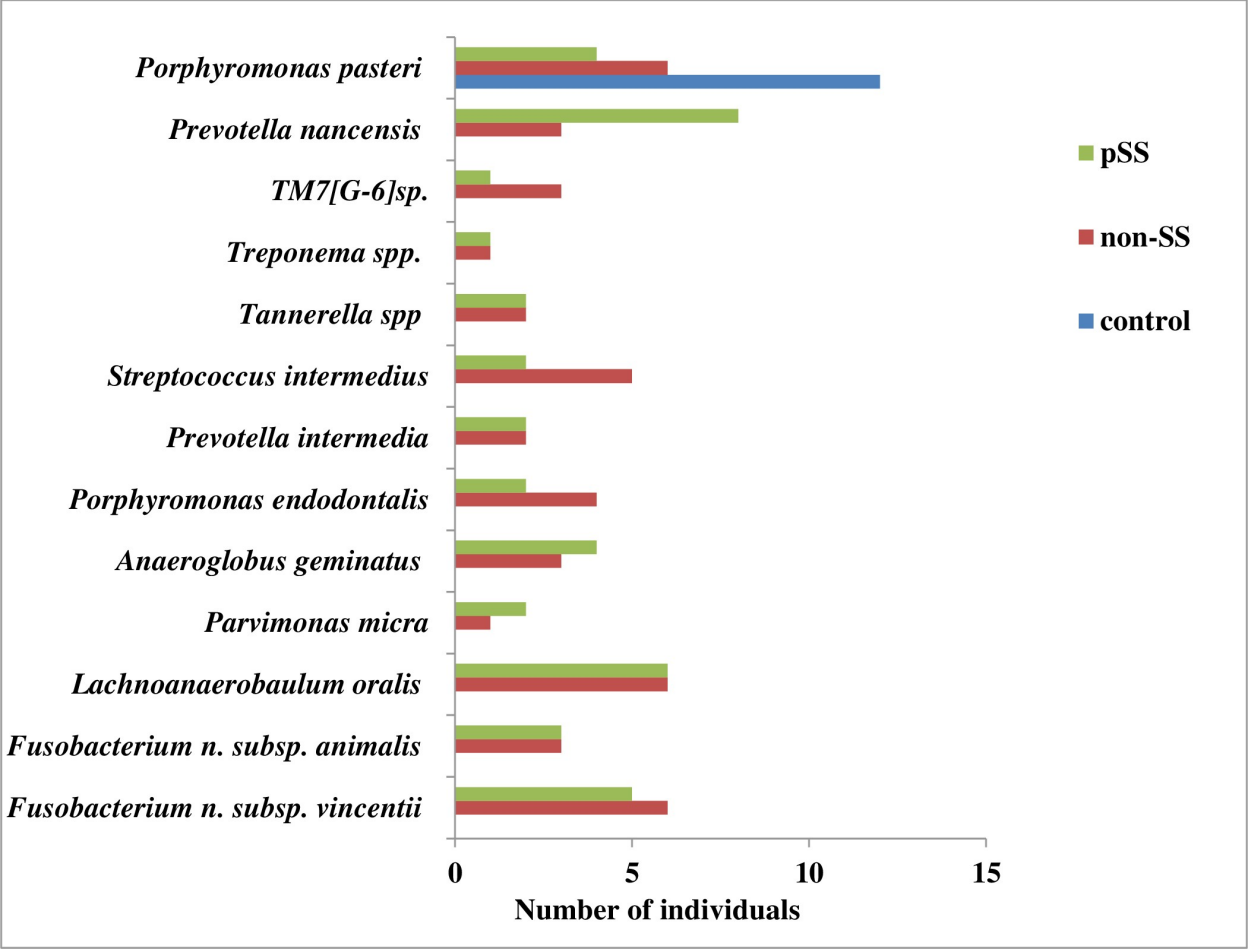
Variable species found in the pSS, non-SS and control groups.

*Porphyromonas pasteri* tended to be present in lower numbers in the pSS group (4 out of 15 samples) and non-SS group (6 out of 15 samples) compared to the control group (12 out of 15 samples). Twelve species from different genera were present only in the pSS and non-SS groups (Fig 4).

**Fig 5:**
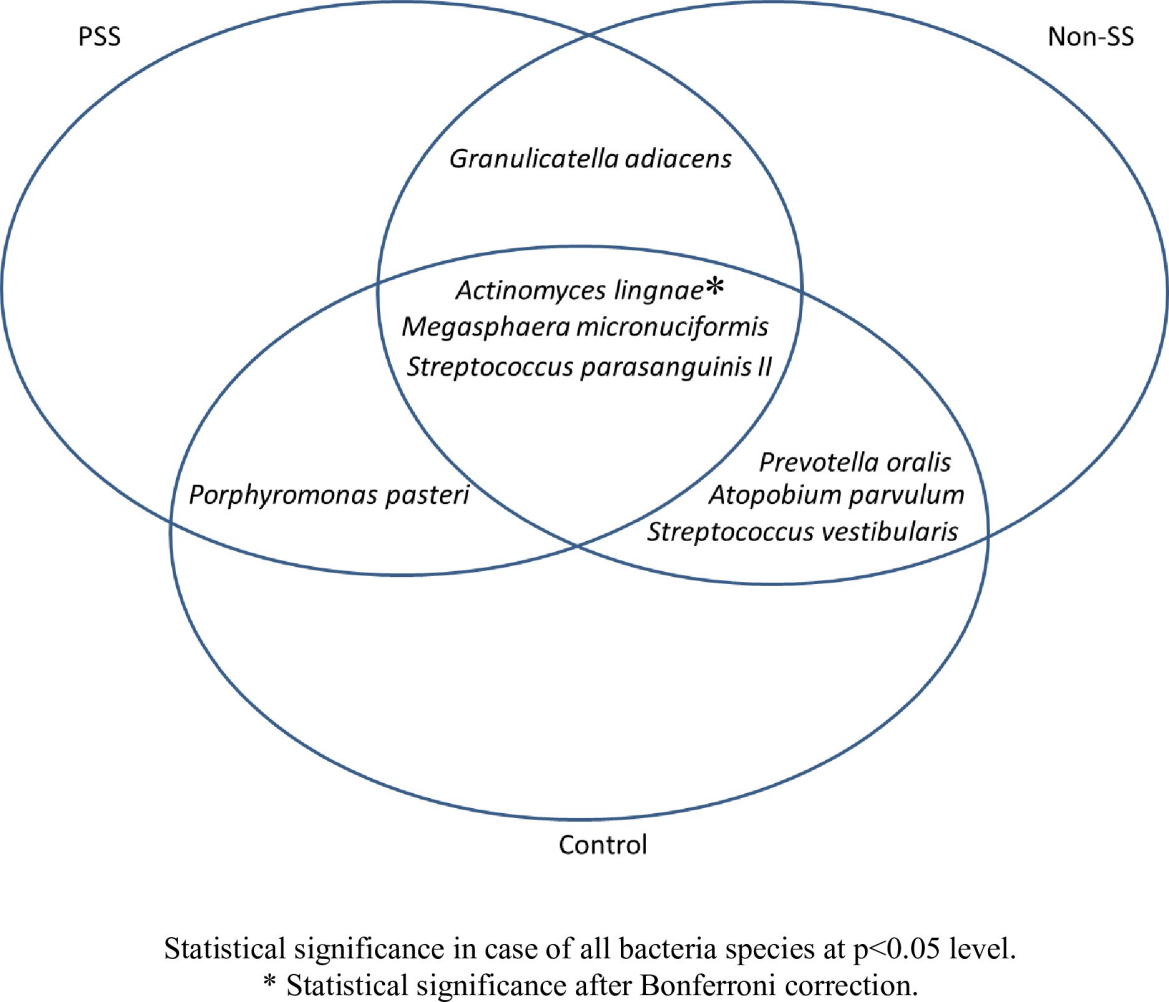
Results of two-sample z-test for the difference between prevalence in the different groups.

As shown in Table 3, the prevalence of 21 bacterial species was found to be significantly different between the three groups. A Z-test showed a statistically significant difference in prevalence of eight species when the three groups were internally compared (Fig 5). Specifically, *Atopobium parvulum* (93.3% vs 40.0%, p = 0.01), *Prevotella oralis* (93.3% vs 53.3%, p = 0.03) and *Streptococcus vestibularis* (100% vs 66.7%, p = 0.017) were more prevalent in the non-SS group than the control group. After Bonferroni correction, this significance was only nearly maintained.

For *Porphyromonas pasteri*, the Z-test showed a significant difference in prevalence between the pSS and control groups (26.7% vs 80%, p = 0.05), but not between the control and non-SS groups. Bonferroni correction resulted in a nearly persistent significance difference between pSS and control groups.

Three species showed significant differences in prevalence between the pSS and control groups and between the non-SS and control groups. There was a statistically significant difference for *Actinomyces lingnae* between the control and non-SS groups (26.7% vs 100%, p = 0.0003). This difference was still significant after Bonferroni correction. However, for this bacteria, the difference was only nearly significant between the control and pSS groups (26.7% vs 66.7%, p = 0.017). *Megasphaera micronuciformis* was significantly different when the control and non-SS groups were compared (26.7% vs 86.7%, p = 0.018), and there was nearly a significant difference in prevalence in this bacteria between the control and pSS groups (26.7% vs 80.0%, p = 0.05). However, these differences were not persistent after Bonferroni correction. *Streptococcus parasanguinis II* was significantly different between the control group and non-SS group (66.7% vs 100%, p = 0.017), but this difference was only nearly significant after Bonferroni correction.

*Granulicatella adiacens* was significantly higher in the non-SS group compared to the pSS group (93.3% vs 46.7%, p = 0.017), and this difference was nearly maintained after Bonferroni correction. No significant differences were detected between the three groups for *Neisseria flavescens*.

When we combined the pSS and non-SS groups and compared subjects with normal salivation (n = 9) to those with hyposalivation (n = 21), we found significant differences in the following four species: *Actinomyces odontolyticus* (44.4% vs 4.8%; p = 0.019), *Campylobacter concisus* (33.3% vs 0.0%; p = 0.021), *Prevotella pallens* (77.8% vs 33.3%; p = 0.025), and *Peptostreptococcaceaex1G1* (44.4% vs 9.52%, p = 0.049).

When we combined the pSS patients and non-SS subjects with normal salivation (n = 9) and compared them with the healthy control group (n = 15) eight species were significantly different. These were *Actinomyces lingnae* (26.7% vs 88.9%, p = 0.009), *Fusobacterium nucleatum subsp vincentii* (0.0% vs 33.3%, p = 0.042), *Lachnoanaerobaculum orale* (0.0% vs 55.6%, P = 0.003), *Megasphaera micronuciformis* (26.7% vs 100.0%, p = 0.001), *Oribacterium asaccharolyticum* (0.0% vs 55.6%, p = 0.003), *Prevotella nanceiensis* (0.0% vs 33.3%, p = 0.042), *Stomatobaculum longum* (0.0% vs 33.3%, p = 0.047), and *Streptococcus intermedius* (0.0% vs 33.3%, p = 0.042). This indicates a dysbiotic shift in both pSS and non-SS patients with normal salivation.

In addition to the analyses described above, patients with hyposalivation in the pSS group (n = 11) were compared to those with hyposalivation in the non-SS group (n = 10). There were five species that differed significantly in abundance between these groups: *Capnocytophaga leadbetteri* (0.00% vs 50.0%, p = 0.012), *Granulicatella adiacens* (36.4% vs 100%, p = 0.004), *Neisseria flavescens* (0.00% vs 40.0%, p = 0.035), *Prevotella nanceiensis* (63.6% vs 10.0%, p = 0.024) and *RuminococcaceaeG1spt* (0.00% vs 40.0%, p = 0.035). After Bonferroni correction, *Actinomyces odontolyticus*, *Campylobacter concisus*, *Actinomyces lingnae*, *Lachnoanaerobaculum orale*, *Megasphaera micronuciformis*, *Oribacterium asaccharolyticum*, *Capnocytophaga leadbetteri*, *Prevotella nanceiensis*, *Granulicatella adiacens*, and *Prevotella nanceiensis* still showed statistically significant differences in abundance.

## Discussion

In this study, the salivary bacterial profile of patients with pSS, dry mouth subjects (non-SS), and age-matched healthy controls was demonstrated using a *16S rRNA* pyrosequencing approach. We found that the salivary bacterial profile of the pSS and non-SS groups differed from the controls. The analysis of the oral microflora at phylum level of the saliva samples showed the existence of nine bacterial phyla, including the same predominant phyla *Firmicutes*, *Bacteroidetes*, *Actinobacteria*, *Proteobacteria*, and *Fusobacteria*, that have been demonstrated in previous studies [9,14].

The genera *Streptococcus*, *Veillonella*, and *Prevotella* were the most abundant in the samples from all groups, confirming that these represent the dominant bacterial genera in saliva [25]. We observed 59 bacterial genera in total for all the samples with 42, 45, and 34 different genera in the pSS, non-SS, and control groups, respectively. This represents a larger diversity than indicated in the work of Siddiqui and co-workers who found 25 genera in a pSS group versus 30 in a non-SS group using V1and V2 hyper variable regions on a Roche 454 GS Junior platform [26]. In another study by Zhou and co-workers (2018), 149 genera were detected in a pSS group compared to 136 in controls [9]. The high number of genera found in that study may be related to their use of a different platform (Illumina Miseq PE300) that is known to return higher reads per sample than that used in our study. Zhou and co-workers (2018) employed the same hypervariable regions (V3-V4) as in our study. However, the sensitivity is expected to be higher using the platform applied in our study since the Roche 454 GS Junior synthetizes longer reads (about 500 nt) than Illumina. Therefore, in our analysis we have been able to identify bacteria at the species level, thus enabling us to reveal specific differences between the study groups [27].

At genus level, the pSS group had a lower abundance of *Neisseria* and *Porphyromonas*, and a higher abundance of *Veillonella*. This is in accordance with Zhou and co-workers (2018), who found a fourfold higher abundance of *Veillonella* in pSS and a lower abundance for *Neisseria* and *Porphyromonas* [9]. Four of the shared dominating genera (*Veillonella*, *Streptococcus*, *Prevotella*, and *Haemophilus*) showed species present in almost all samples in the three groups.

The high prevalence of *Porphyromonas pasteri* in the healthy controls in our study was in agreement with the results by Yasunaga and co-workers (2017) [28]. They found *P*. *pasteri* to be associated with good dental health in the saliva of 139 individuals [28,29]. Furthermore, in our study, twelve species were detected only in the pSS and non-SS groups. Of these, important periodontal species were *P*. *intermedia*, *F*. *nucleatum vincentii*, *P*. *micra*, *S*. *intermedius*, and *P*. *endodontalis* as well as *Treponema spp* and *Tannerella spp*. This finding may indicate signs of dysbiosis in the pSS and non-SS groups.

One species, *G*. *adiacens*, had a significantly lower prevalence among the pSS patients compared to the non-SS group. Lourenco and co-workers (2014) found more *G*. *adiacens* in a healthy control group. This could support our findings that in pSS there is a shift in the composition of the oral bacterial flora [30].

All these bacterial species are commonly found in saliva, and their mutual relationships are dependent on local host factors such as diet and salivary pH. The significantly different abundances of the bacteria in the three groups included in our study will therefore, also depend on various other host factors. Zaura and co-workers (2017) described how different saliva microbiota clusters represent different ecological properties and various levels of specialization [12]. Specialization in amino acid fermentation results in an elevated salivary pH and increased production of bacterial deaminases and proteases that induces inflammation. The more specialized the ecosystem becomes, the more it may shift toward dysbiosis. *S*. *salivarius* is linked to a saccharolytic life style whereas *Megasphaera micronuciformis* and *Prevotella oralis* are linked to a proteolytic lifestyle [12].

The presence of secondary colonizer bacteria such as *P*. *intermedia*, *F*. *nucleatum*, and *P*. *endodontalis* tended to be slightly increased in the non-SS and pSS groups compared to the controls when using primers for the V3-V5 hypervariable region. This may indicate a dysbiosis in the saliva of our pSS and non-SS groups. A similar finding has been shown in SS patients (with or without reduced salivation) in other studies [14,26]. A recent study that used primers for the V4 hypervariable region demonstrated dysbiosis in the buccal microbiome in both pSS and non-SS patients further strengthen our results [9,14].

Although our study groups were of limited size, the strength of our study lies in the analysis of the sequencing results down to species level. Furthermore, we were able to observe significant differences between the sample sets using several statistical approaches. Our findings are further supported by the comparable, but somewhat larger study of van der Meulen et al [14], in which similar observations as those found in our work were made at genus level.

The results of this study suggest that hyposalivation alone is not necessarily the cause of the observed dysbiosis in pSS and non-SS. Accordingly, several studies including this study, indicate that microbiome investigations of the oral cavity are important [31,32]. The results of such studies will be of value in the diagnosis and identification of autoimmune diseases], and the current results may be a step towards the identification of early, non-invasive diagnostic biomarkers for pSS.
